# Gallstones and Diseases of the Bile-Ducts

**Published:** 1907-06

**Authors:** 


**Gallstones and Diseases of the Bile-Ducts.** By J. BLAND-SUTTON, F.R.C.S. Pp. vi., 233. London: James Nisbet & Co., Limited. 1907.

The book consists of a series of lectures delivered at the Middlesex Hospital. The various subjects are treated in a comparatively brief but lucid manner, and the work is not meant to compete with the larger monographs on these diseases.

It is a useful compendium for the student and practitioner rather than the operating surgeon. The indications for treatment are on orthodox lines, but the author holds strong views on the desirability of performing cholecystectomy as a routine measure. This again is in accordance with the views of many surgeons, but there are arguments against, as well as for, this procedure. Because gallstones are recognised as a predisposing cause of cancer we are told that "it behoves surgeons when removing gallstones to excise the gall bladder;" but logically the removal of the stones is surely the essential need, and the desirability (if it exists) of performing cholecystectomy rests on other grounds.

There are some good illustrations and a few references at the end of the chapters.

